# Regulation of Serotonin-Induced Trafficking and Migration of Eosinophils

**DOI:** 10.1371/journal.pone.0054840

**Published:** 2013-01-23

**Authors:** Bit Na Kang, Sung Gil Ha, Nooshin S. Bahaie, M. Reza Hosseinkhani, Xiao Na Ge, Malcolm N. Blumenthal, Savita P. Rao, P. Sriramarao

**Affiliations:** 1 Laboratory of Allergic Diseases and Inflammation, Department of Veterinary and Biomedical Sciences, University of Minnesota, St. Paul, Minnesota, United States of America; 2 Department of Medicine, University of Minnesota, St. Paul, Minnesota, United States of America; Fundação Oswaldo Cruz, Brazil

## Abstract

Association of the neurotransmitter serotonin (5-HT) with the pathogenesis of allergic asthma is well recognized and its role as a chemoattractant for eosinophils (Eos) *in vitro* and *in vivo* has been previously demonstrated. Here we have examined the regulation of 5-HT-induced human and murine Eos trafficking and migration at a cellular and molecular level. Eos from allergic donors and bone marrow-derived murine Eos (BM-Eos) were found to predominantly express the 5-HT2A receptor. Exposure to 5-HT or 2,5-dimethoxy-4-iodoamphetamine (DOI), a 5-HT2A/C selective agonist, induced rolling of human Eos and AML14.3D10 human Eos-like cells on vascular cell adhesion molecule (VCAM)-1 under conditions of flow *in vitro* coupled with distinct cytoskeletal and cell shape changes as well as phosphorylation of MAPK. Blockade of 5-HT2A or of ROCK MAPK, PI3K, PKC and calmodulin, but not G_αi_-proteins, with specific inhibitors inhibited DOI-induced rolling, actin polymerization and changes in morphology of VCAM-1-adherent AML14.3D10 cells. More extensive studies with murine BM-Eos demonstrated the role of 5-HT in promoting rolling *in vivo* within inflamed post-capillary venules of the mouse cremaster microcirculation and confirmed that down-stream signaling of 5-HT2A activation involves ROCK, MAPK, PI3K, PKC and calmodulin similar to AML14.3D10 cells. DOI-induced migration of BM-Eos is also dependent on these signaling molecules and requires Ca^2+^. Further, activation of 5-HT2A with DOI led to an increase in intracellular Ca^2+^ levels in murine BM-Eos. Overall, these data demonstrate that 5-HT (or DOI)/5-HT2A interaction regulates Eos trafficking and migration by promoting actin polymerization associated with changes in cell shape/morphology that favor cellular trafficking and recruitment via activation of specific intracellular signaling molecules (ROCK, MAPK, PI3K and the PKC-calmodulin pathway).

## Introduction

5-Hydroxytryptamine (5-HT, serotonin) is one of the most extensively studied neurotransmitters of the central nervous system (CNS) which is known to have a multitude of physiological functions outside the CNS. These include stimulation of cytokine [1], [2], [3], [4] and chemokine production [4], [5], vasoconstriction [6], tissue regeneration [7], cell (fibroblasts, smooth muscle cells [SMC], endothelial cells) proliferation [8], [9], [10] and migration (eosinophils [Eos], mast cells [MC], SMC, dendritic cells [DC]) [4], [11], [12], [13], and regulation of the immune system [14]. 5-HT is known to exert its effects by binding to cell surface receptors which are classified into seven distinct families (5-HT1 to 5-HT7) comprising 14 distinct subtypes based on their structural diversity and mode of action [15], [16]. The effects of 5-HT on inflammatory cells are largely mediated by one or more of the following receptors, 5-HT1A [12], [17], [18], [19], 5-HT2A [8], [9], [11], 5-HT3 [4], [20], 5-HT4 [1], [2], [13] and 5-HT7 [1], [2].

In addition to its functions described above, there is substantial evidence indicating a role for 5-HT in the pathophysiology of asthma. Symptomatic asthmatic patients have increased plasma 5-HT levels that correlate positively with clinical status and negatively with pulmonary function [21]. Clinical studies using the 5-HT uptake-enhancing drug tianeptine have demonstrated a dramatic and sudden decrease in both clinical rating and free plasma 5-HT levels with increased pulmonary function in children [22]. More recent studies have demonstrated that 5-HT inhibits IL-12 and induces PGE2 production by alveolar macrophages (AM) thus modulating the cytokine network in the lung and contributing to the pathogenesis of asthma (by reducing Th1 cytokines) [3]. During allergic airway inflammation and asthma, Eos are the predominant inflammatory cells recruited to the lungs [23]. We have previously demonstrated that 5-HT functions as a potent chemoattractant for human Eos [11]. In addition, our studies as well as those by other investigators have shown that 5-HT promotes the development of allergic airway inflammation, airway hyperresponsiveness (AHR) and remodeling in murine models of asthma [11], [24], [25]. While our previous studies have demonstrated that 5-HT promotes Eos migration and recruitment, the regulation of these 5-HT-mediated events is not known. In the current study, we have used a systematic approach enabling interpretation of cross-species findings between human and murine Eos to examine how 5-HT regulates various aspects of Eos trafficking (rolling and adhesion *in vitro* and *in vivo* within inflamed blood vessels) and migration including the role of specific signaling molecules involved in these events.

## Materials and Methods

### Ethics Statement

All studies involving mice were performed following standards and procedures approved by the Institutional Animal Care and Use Committee at the University of Minnesota.

### Animals

BALB/c mice, 8 weeks of age (Charles River, Wilmington, MA) maintained under standard pathogen free conditions were used.

### Culture of AML13.4D10 cells

The Eos-like cell line AML14.3D10 (kind gift from Cassandra C. Paul, Wright State University School of Medicine, Dayton, OH) used as a model cell line for the study of human Eos was maintained in RPMI-1640 supplemented with 8% fetal bovine serum, 2 mM L-glutamine, 1 mM sodium pyruvate, and β-mercaptoethanol (5×10^–5^ M) as described previously [26].

### Human and murine Eos

Human Eos were isolated from peripheral blood of donors with a clinical diagnosis of asthma and/or rhinitis who had elevated peripheral blood Eos ranging between 7–18% as described previously [27]. Briefly, granulocytes collected from peripheral blood by Ficoll-Paque (GE Healthcare, Piscataway, NJ) centrifugation were treated with ammonium chloride solution (150 mM ammonium chloride, 10 mM potassium bicarbonate, 0.5 M EDTA, pH 8.0) to remove red blood cells and Eos were enriched based on negative selection using EasySep® Eos enrichment kits (Stemcell Technologies, Vancouver, BC, Canada) according to the manufacturer's protocol. Viability and purity were routinely found to be >95%.

Eos were cultured from BM of BALB/c mice as previously described [27], [28]. Eos differentiation was confirmed based on Hema 3 staining (Thermo Fisher Scientific Co., Pittsburgh, PA) of cytocentrifuged BM cultures and expression of Eos-specific major basic protein (MBP) by confocal microscopy with rat mAb against murine MBP (2.5 μg/ml, provided by Dr. J. Lee, Mayo Clinic Arizona, Scottsdale, AZ) using a FLUOVIEW FV1000/BX61 – Confocal Laser Scanning Biological Microscope equipped with an UPlanSApo lens (20×/0.85 [oil]) and a PlanApo N lens (60×/1.42 [oil]). FV10-ASW 2.0 software was used for image acquisition (Olympus, Melville, NY). In addition, expression of Siglec-F was assessed by flow cytometry using PE-conjugated rat anti mouse Siglec-F (5 µg/ml, BD Biosciences, San Diego, CA) with a FACScan flow cytometer (BD Biosciences) and FlowJo software (version 7.1, Tree Star, Ashland, OR). Differentiated cells between day 11–13 of culture that were 99% Hema 3 positive and expressed both MBP and Siglec-F were used in studies.

### Expression of 5-HT receptors by RT-PCR

Extraction of total RNA from human Eos (3 – 4×10^6^), AML14.3D10 cells (5×10^6^) and murine BM-Eos (5×10^6^) followed by cDNA synthesis was carried out as described in our previous studies [29]. cDNA from human Eos and AML14.3D10 cells was amplified with primers specific for human 5-HT1A, 5-HT1B, 5-HT1E, 5-HT2A, 5-HT2B, 5-HT2C, 5-HT3, 5-HT4, 5-HT6 or 5-HT7 [12]. Expression of 5-HT2 receptors by murine BM-Eos was evaluated using previously described primers specific for murine 5-HT2A, 5-HT2B and 5-HT2C [5]. Expression of β-actin was monitored as an internal control for both human [12] and murine Eos [30]. PCR amplification was performed in a DNA Engine® PTC-0200 cycler (Bio-Rad Laboratories, Hercules, CA) under the following conditions: initial denaturation at 94°C for 5 min followed by 30 cycles of amplification (94°C for 15 *s* [denature], 60°C for 30 *s* [anneal] and 68°C for 2 min [extension]), and a final extension at 68°C for 5 min. PCR products were separated on 2% agarose gels, stained with ethidium bromide and visualized using the FluorChem HD2 imaging system (Alpha Innotech, San Leandro, CA).

### Assessment of Eos rolling in vitro

Rolling of human Eos, AML14.3D10 cells or murine BM-Eos on vascular cell adhesion molecule (VCAM)-1 under conditions of flow was evaluated in an *in vitro* parallel plate flow chamber as described previously [31], [32]. Cells (2×10^5^/ml) were incubated with 10 μM 5-HT (Sigma-Aldrich Corp., St. Louis, MO) or 2,5-dimethoxy-4-iodoamphetamine (DOI), a 5-HT2A/2C agonist (Sigma-Aldrich Corp.) [33], [34], [35], for 5 min prior to infusion into the flow chamber at a flow rate of 1 ml/min (wall shear stress, 1.0–2.0 dynes/cm^2^). Interaction of the infused cells with recombinant human (rh) or recombinant murine (rm) VCAM-1 (Leinco Technologies, Inc., St. Louis, MO)-coated cover-slips was analyzed as described previously [31]. In experiments to identify the involvement of 5-HT2A receptors in promoting DOI-induced rolling, cells were incubated with MDL-100907 (5-HT2A antagonist) [36], [37], [38] at a concentration of 10 μM based on initial dose response (1–100 µM) studies performed to identify the optimal dose (data not shown) and then exposed to DOI for 5 min prior to infusion into the flow chamber. To examine the role of specific signaling molecules in mediating DOI-induced rolling, Eos were incubated with Y27632 (Rho-associated protein kinase [ROCK] inhibitor, Sigma-Aldrich Corp.), PD98059 (MAPK inhibitor, Sigma-Aldrich Corp.), LY294002 (phosphoinositide 3 kinase [PI3K] inhibitor, Sigma-Aldrich Corp.), pertussis toxin (PT, G_αi_ inhibitor, Sigma-Aldrich Corp.), bisindolylmaleimide-1 (BIM[I], PKC inhibitor, Calbiochem, La Jolla, CA), trifluoperazine hydrochloride (TFP, calmodulin antagonist, Sigma-Aldrich Corp.) or DMSO (Sigma-Aldrich Corp.) alone (vehicle) for 20 min and then exposed to DOI. PT was used at a concentration of 100 ng/ml, while all other inhibitors were used at 10 μM. Results were expressed as the number of rolling cells/min.

### Western blot analysis

To evaluate the effect of DOI on activation of p-44/42 MAPK, AML14.3D10 cells (5×10^6^) were pre-treated with DMSO alone (vehicle) or 10 µM PD98059 and then exposed to DOI as described above. Cells were immediately centrifuged, lysed in a mixture of 5× SDS-PAGE sample buffer and RIPA buffer containing protease and phosphatase inhibitor cocktail (Sigma-Aldrich, St. Louis, MO), electrophoresed (12% polyacrylamide gels), transferred to PVDF membranes (Millipore, Bedford, MA), blocked (Odyssey Blocking buffer, LI-COR Biosciences, Co., Lincoln, NE) and incubated overnight at 4°C with primary antibodies against p44/42 MAPK (0.01 µg/ml, Cell Signaling Technology, Danvers, MA) or phospho p44/42 MAPK (0.15 µg/ml, Cell Signaling Technology). Anti-β-actin antibodies (1∶5000; BD Biosciences) were used to monitor levels of β-actin expression as an internal control. IRDye−800CW anti-rabbit or IRDye-680 anti-mouse IgG (1∶5000, LI-COR Biosciences) was used as the secondary antibody and detection was carried out with an Odyssey Infrared Imaging System (LI-COR Biosciences).

### Chemotaxis assay

Migration of murine BM-Eos (5×10^4^ cells/well) in response to 5-HT or DOI (10 μM) or medium alone was evaluated using 96-well Transwell® Chambers (5.0 µm) as described previously [27]. To assess the involvement of various signaling molecules during DOI-induced migration, cells were pre-treated with specific inhibitors or DMSO alone as described earlier prior to addition to Transwell® Chambers. Eos migration was evaluated after 4 h at 37°C using an Olympus CK2 inverted microscope with a 40× objective. Cells in a fixed number of randomly selected non-overlapping fields were counted for each well for each experiment. The assay was performed 3 (for PT, BIM[I] and TFP) or 5 (all other inhibitors) times in triplicate. The average number of cells/field/well was determined and results were expressed as percentage of background migration observed in wells containing medium alone. For the inhibition studies, results were expressed as a percentage of the migration observed towards DOI.

To examine the role of Ca^2+^ in DOI-induced migration of Eos, assays were carried out in HBSS with and without Ca^2+^ and Mg^2+^ as described above. To deplete intracellular Ca^2+^, Eos suspended in HBSS without Ca^2+^ and Mg^2+^ were pre-incubated with 20 µM BAPTA/AM [39](Sigma-Aldrich Corp.) or DMSO (vehicle) for 20 min prior to assay.

### Assessment of changes in cell shape and morphology

Human Eos shape change was examined using the gated autofluorescence/forward scatter (GAFS) assay as previously described [40]. Briefly, human polymorphonuclear leukocytes (PMNLs) from peripheral blood collected by Percoll gradient centrifugation [41] were washed (PBS containing 0.1% BSA, 10 mM glucose, 10 mM HEPES with Ca^2+^ Mg^2+^) and resuspended at 1×10^7^ cells/ml. After an initial incubation for 30 min at 37°C, cells (2.5×10^6^) were stimulated with 5-HT (10 μM) or C5a (1 nM, positive control) for 1 or 10 min at 37°C, fixed (0.25% paraformaldehyde in PBS) at 4°C to maintain cell shape and analyzed immediately by flow cytometry. Eos were identified and gated by their natural autofluorescence, which is greater than that of neutrophils in the fluorescence channel FL-2. Data for events within this gated region (Eos) representing both responding as well as non-responding cells were acquired and results were expressed as percent increase in mean forward scatter (FSC) of 5-HT-stimulated cells compared to unstimulated cells.

Changes in cell morphology/cytoskeleton after Eos adhesion were evaluated as described in our recent studies [29]. Cells (5×10^4^ cell) were first allowed to attach to rh or rm VCAM-1-coated (10 μg/ml) cover slips in PBS containing inhibitors or DMSO alone (vehicle control) and then exposed to 5-HT or DOI for 5 min. Cover slips were washed with PBS and adhered cells were evaluated by confocal microscopy after FITC-phalloidin staining (Invitrogen, Carlsbad, CA). In some experiments, cells were counter-stained with 2 μg/ml DAPI (Roche Applied Science, Indianapolis, IN) for 5 min at room temperature to visualize the nucleus. Differences in cell morphology between vehicle- and antagonist/inhibitor-treated cells after exposure to DOI was assessed as described previously [27]. The number of adherent cells in a fixed number of randomly selected non-overlapping fields of each cover-slip in each experiment was manually counted and the number of cells exhibiting shape changes (cell spreading with membrane protrusions i.e., leading edges, lamellipodia or filopodia from a round cell body) were identified and expressed as a percentage of the total number of adhered cells in the field.

Actin polymerization was assessed based on phalloidin binding using FITC-phalloidin (Invitrogen) followed by flow cytometry as previously described [27]. AML14.3D10 cells (1×10^6^ cells) were pre-treated with antagonist/inhibitors or DMSO alone before exposure to DOI for 5 min at 37°C. Total cellular filamentous actin (F-actin) was evaluated by flow cytometry after staining with FITC-conjugated phalloidin. A total of 10,000 events were acquired, and results were expressed as percent change in cell fluorescence relative to untreated cells.

### Measurement of intracellular calcium [Ca^2+^]_i_ levels

[Ca^2+^]*_i_* in murine BM-Eos was determined using the permeant Ca^2+^ indicator dye Fura-2 AM (Molecular Probes, Invitrogen) as described previously [32]. Fura-2 AM-loaded cells were alternately excited at 340 and 380 nm with a Lambda DG-4 high speed wavelength switcher (Sutter Instrument Co., Novato, CA). Basal [Ca^2+^]*_i_* was measured by real-time digital video fluorescence imaging using NIS-Elements imaging software (Nikon Instruments, Inc.) for 1 min. Cells were then stimulated with 10 μM DOI and evaluated for an additional 5 min to assess agonist-induced [Ca^2+^]_i_ responses. Cells were finally exposed to 2 μM 4-Bromo-calcium Ionophore (Sigma Aldrich Corp.) as a positive control and monitored for 2 min.

### Eos trafficking in vivo by intravital microscopy

Effect of 5-HT on murine Eos trafficking *in vivo* was assessed by intravital microscopy (IVM) as described in our recent studies [29]. In brief, the cremaster muscle microcirculation of anesthetized male BALB/c mice was exteriorized approximately 5 h after intrascrotal injection of TNFα. Carboxyfluorescein succinimidyl ester (CFSE, Invitrogen)-labeled murine BM-Eos (1×10^7^) were administered via the carotid artery to the prepared animal placed on the stage of an intravital microscope (Leica Microsystems Inc., Bannockburn, IL) and adhesive interactions of the labeled cells with cremaster muscle post capillary venules was evaluated and digitally recorded (baseline). The exposed cremaster microcirculation was superfused with 1 ml of 100 nM 5-HT or saline (as control) after 20–30 min and 5-HT-induced interactions of circulating labeled Eos within the microvessels was evaluated. This was followed by off-line analysis of recorded digital images to determine the number of rolling and adherent cells as well as the velocity of rolling Eos as described in our previous studies [42], [43]. The number of recorded fields analyzed per mouse was 16±2 (mean ± SEM) which included 1–2 vessels per field. The number of rolling cells was expressed as rolling fraction, which was a percentage of the total number of cells passing through the same reference point. Adherent cells were defined as those cells remaining stationary for at least 1 min and expressed as the number of adherent cells/500 µm length of blood vessel. The velocity of rolling Eos was determined by measuring the time taken for the cells to travel between two reference points and is expressed as µm/sec.

### Expression of adhesion molecules

Murine BM-Eos were treated with 10 μM DOI or PBS (control) for 5 min at 37°C. After washing cells with PBS, expression of α4 (CD49d), LFA-1 (CD11a), and Mac-1 (CD11b) was assessed by flow cytometry using rat mAbs against murine α4 (PS/2), CD11a (eBiosciences, San Diego, CA), and CD11b (eBiosciences) respectively, followed by FITC-conjugated goat anti-rat IgG as the secondary antibody. Depending on the mAb, rat IgG2a or 2b was used as the isotype matched control. Expression of L-selectin (CD62L) was evaluated using FITC-conjugated anti-mouse CD62L (BD Biosciences) with FITC-conjugated rat-IgG2a as the isotype control. All antibodies were used at a final concentration of 5 µg/ml.

### Statistical analysis

Results are expressed as the mean ± SEM. Statistical significance was determined using the Student's *t*-test. A p value <0.05 was considered as significant.

## Results

### 5-HT induces trafficking of human Eos

**Figure 1:**
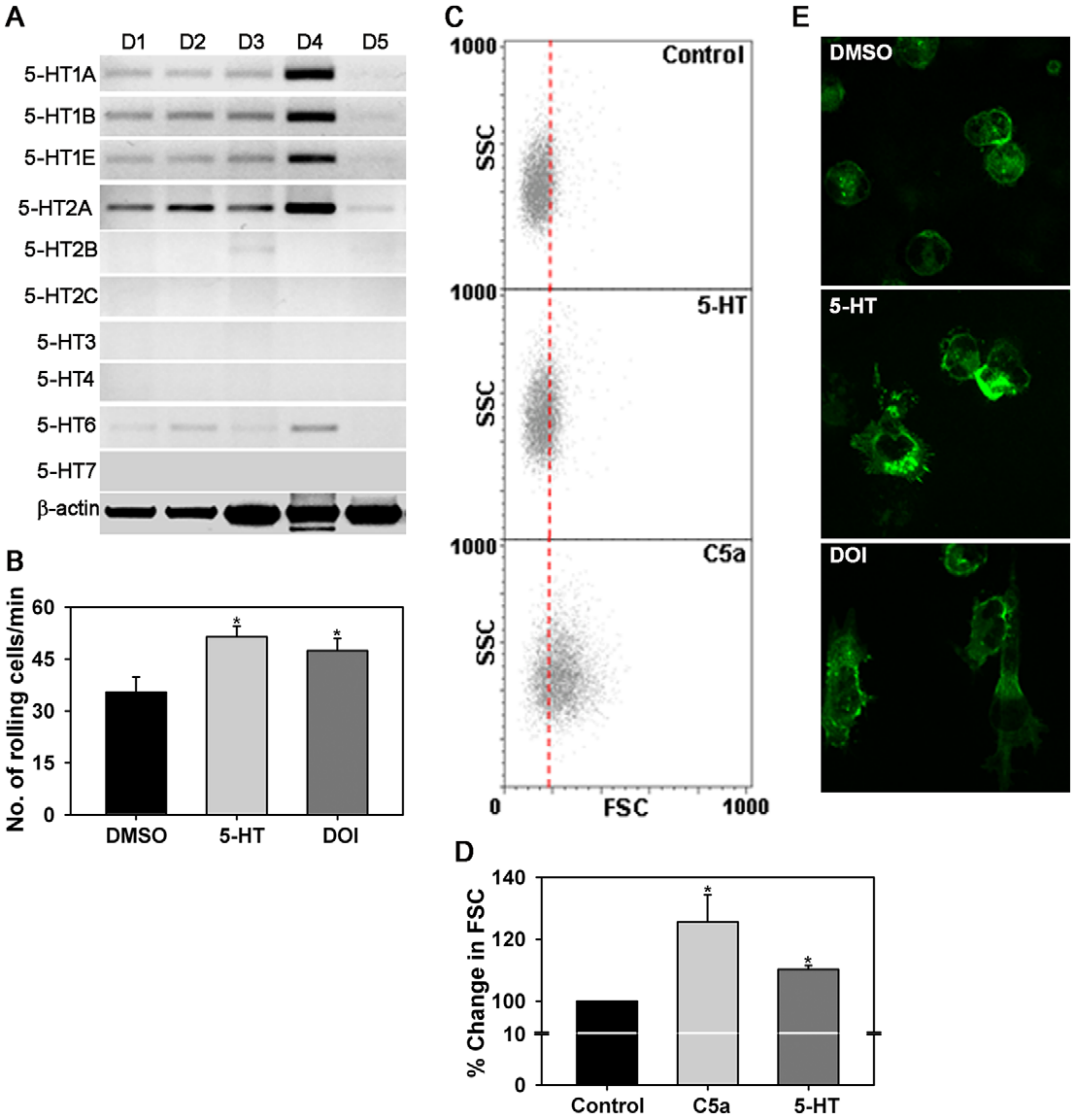
5-HT induces rolling of human Eos and alters cell shape and morphology. (**A**) Expression of 5-HT receptors by human Eos from allergic donors (D1-D5) by RT-PCR. Expression of β-actin is shown as the internal control. (**B**) Rolling of human Eos on rh VCAM-1-coated cover-slips under conditions of flow *in vitro*. Cells were treated with 5-HT or the 5-HT2A/2C receptor agonist DOI (both at 10 µM) for 5 min prior to infusion into the flow chamber. Cells treated with DMSO (vehicle) alone served as a control. (**C**) FSC versus side scatter (SSC) dot plots for untreated (control) and 5-HT (10 µM)- or C5a (1 nM, positive control)-treated human Eos after 1 min by GAFS assay. (**D**) Human Eos shape change measured as an increase in FSC after treatment with 5-HT or C5a for 10 min at 37°C measured. Results are expressed as percent change in mean FSC of stimulated cells compared to control cells. (**E**) Confocal microscopy of FITC-phalloidin stained human Eos initially allowed to attach to rh VCAM-1 on cover-slips for 20 min in PBS followed by an additional 5 min in the presence of 5-HT or DOI (both at 10 µM) or vehicle. Magnification ×600. Combined data (Mean ± SEM) of Eos from 4–6 donors is shown for B and D. *p<0.01 for 5-HT and <0.03 for DOI in B for comparison with DMSO-treated Eos and <0.01 in D for comparison with control Eos. Representative data of six independent experiments in C and three independent experiments in E with Eos from different donors is shown.

A role for 5-HT in promoting human Eos migration *in vitro* has previously been shown [11]. Here, we evaluated the expression of various 5-HT receptors by Eos from donors with asthma and/or rhinitis (n = 5) by RT-PCR with primers specific for each subtype and found that human Eos express 5-HT1A, 5-HT1B, 5-HT1E, 5-HT2A, 5-HT2B and 5-HT6 receptors with 5-HT2A being the most predominantly expressed, albeit at variable levels among the different donors (Fig. 1, A). Expression of 5-HT2C, 5-HT3, 5-HT4 and 5-HT7 was not detected. Next, the effect of 5-HT and the 5-HT2A/2C receptor agonist DOI on human Eos rolling as well as cell shape and morphology that promote directional movement during cell trafficking was investigated. Human Eos treated with 5-HT or DOI for 5 min exhibited significantly increased rolling on rh VCAM-1 under conditions of flow *in vitro* (Fig. 1, B). GAFS assay indicated that human Eos exposed to 5-HT undergo shape change detected as an increase in mean forward scatter (FSC) compared to untreated (control) cells at 1 min that was present even at 10 min. As expected, an increase in FSC was also observed when Eos were exposed to C5a, the positive control. To represent both time points, dot plots for Eos shape change at 1 min for a representative donor as well as increase in FSC observed at 10 min for all donors tested are shown in Figs. 1, C and D. This effect of 5-HT was further substantiated with confocal microscopy studies which demonstrated that treatment of human Eos adherent on VCAM-1 with 5-HT or DOI induces distinct changes in the cytoskeleton and cell morphology compared to cells treated with vehicle alone (Fig. 1, E). While vehicle-treated cells adhered to VCAM-1 were mostly round, several 5-HT- or DOI-treated cells exhibited formation of distinct leading edges with lamellipodia and filopodia. In addition, most of the control cells displayed phalloidin binding at the cell periphery/margin and in the center or main body of the cell, while a large number of the cells treated with 5-HT or DOI demonstrated more intense phalloidin binding at the leading edges with less in the center of the cell.

### Effect of 5-HT and DOI on AML14.3D10 cells

**Figure 2:**
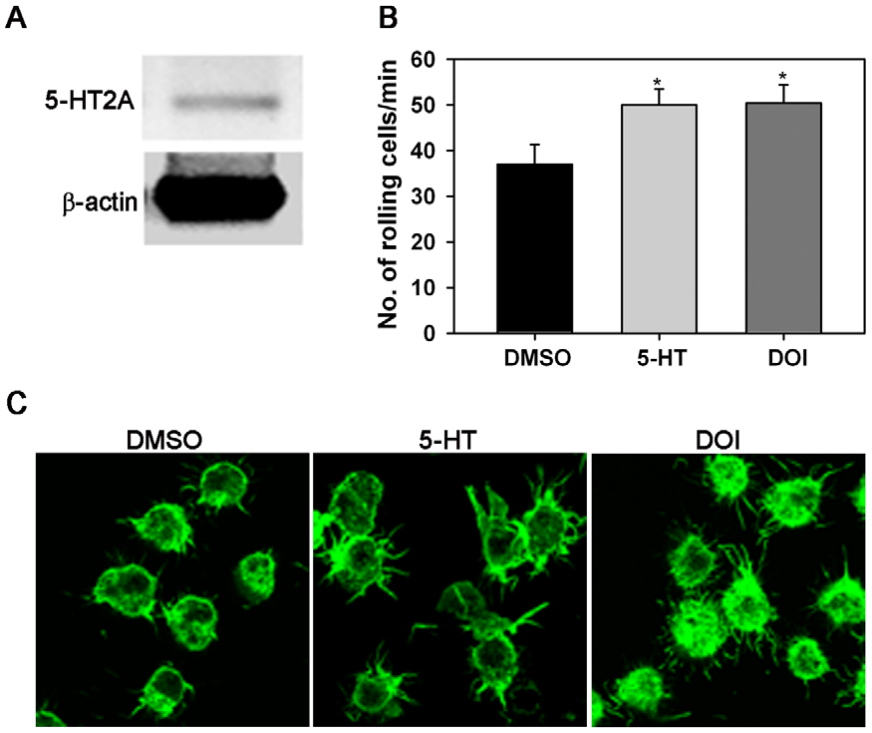
5-HT and DOI induce rolling of AML14.3D10 human Eos-like cells. (**A**) Expression of 5-HT2A by AML14.3D10 cells by RT-PCR with β-actin as the internal control. (**B**) Rolling of AML14.3D10 cells on rh VCAM-1-coated cover-slips under conditions of flow *in vitro* after treatment with 5-HT, DOI or DMSO (vehicle) for 5 min prior to infusion into flow chamber. (**C**) Confocal microscopy of FITC-phalloidin stained AML14.3D10 cells adhered to rh VCAM-1 on cover-slips in the presence of vehicle (DMSO), 5-HT or DOI as described in Figure 1. Magnification ×600. Combined data (Mean ± SEM) of 3 (for 5-HT)-5 (for DOI) independent experiments in duplicate is shown for B. *p<0.03 in B for comparison of 5-HT- or DOI-treated versus vehicle-treated cells. Data shown in C is representative of three independent experiments.

Our previous studies with human Eos have demonstrated that 5-HT-induced migration is mediated via 5-HT2A [11]. Given the donor-to-donor variability observed in 5-HT2A expression by human Eos (Fig. 1, A), we opted to use AML14.3D10 cells, a human Eos-like cell line, to further investigate the role of 5-HT/5-HT2A interactions. We first established that AML14.3D10 cells express 5-HT2A by RT-PCR (Fig. 2, A). Next, the ability of 5-HT and DOI to induce rolling of AML14.3D10 cells on VCAM-1 under conditions of flow was confirmed (Fig. 2, B). Further, similar to Eos from allergic donors, both 5-HT and DOI induced changes in cytoskeleton/morphology of AML14.3D10 cells adherent on VCAM-1 with treated cells exhibiting multiple well defined filopodia relative to cells treated with DMSO (Fig. 2, C). Not only do these studies confirm our observations with Eos from allergic donors, but also demonstrate that AML14.3D10 cells can be used as a model system representative of human Eos to evaluate the effects of 5-HT or DOI.

### DOI-induced rolling and changes in morphology of AML14.3D10 cells involves ROCK, MAPK and PI3K signaling

**Figure 3:**
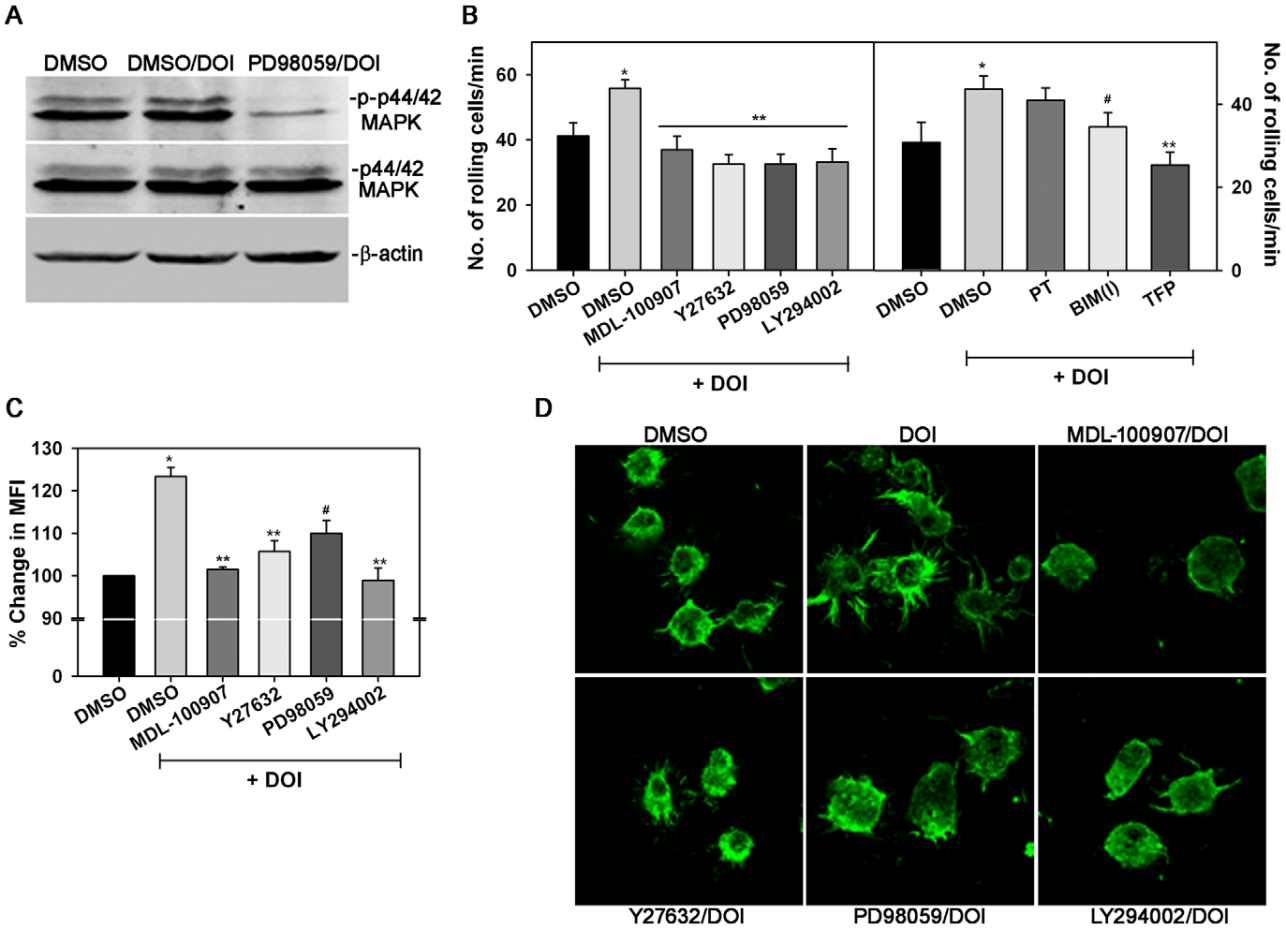
DOI-induced rolling of AML14.3D10 cells involves activation of ROCK, MAPK PI3K, PKC and calmodulin. (**A**) Phosphorylation of p-44/42 MAPK in cells exposed to DOI after pre-treatment with PD98059 (10 µM, MAPK inhibitor) or DMSO (vehicle for inhibitor) for 5 min. Cell lysates were analyzed by Western blot analysis. (**B**) DOI-induced rolling of AML14.3D10 cells on rh VCAM-1-coated cover-slips under conditions of flow *in vitro* after treatment with MDL-100907 (5-HT2A antagonist), Y27632 (ROCK inhibitor), PD98059, LY294002 (PI3K inhibitor) (left panel) or PT (G_αi_ inhibitor), BIM(I) (PKC inhibitor), TFP (calmodulin inhibitor) (right panel). Cells treated with DMSO alone served as the control. All inhibitors were used at a concentration of 10 µM, except PT which was used at 100 ng/ml, for 20 min and then exposed to DOI for 5 min prior to infusion into the flow chamber. (**C**) Total F-actin measured by flow cytometry after FITC-phalloidin staining of AML14.3D10 cells treated with MDL-100907, Y27632, PD98059, LY294002 or vehicle for 20 min and then exposed to DOI. Results are expressed as percent change in mean fluorescence intensity (MFI) relative to vehicle-treated cells. (**D**) FITC-phalloidin staining and confocal microscopy of AML14.3D10 cells adhered to rh VCAM-1-coated cover-slips in the presence of MDL-100907, Y27632, PD98059, LY294002 or vehicle and then exposed to DOI (as described in Figure 1). Magnification ×600. Combined data (Mean ± SEM) of four independent experiments in B and three independent experiments in C is shown. *p<0.03 for comparison of cells exposed to DOI versus vehicle. **p<0.01 and ^#^p<0.05 for comparison of cells exposed to DOI in the presence of inhibitor versus cells exposed to DOI in the presence of vehicle. Data shown in D is representative of two independent experiments.

Exposure of AML14.3D10 cells to DOI for 5 min resulted in phosphorylation of p-44/42 MAPK which was inhibited when cells were treated with PD98059 prior to exposure to DOI (Fig. 3, A). Next, we evaluated DOI-induced rolling of AML14.3D10 cells on VCAM-1 under conditions of flow in the presence of various inhibitors. Pretreatment of cells with MDL-100907 inhibited DOI-induced rolling of AML14.3D10 cells to background levels confirming the involvement of 5-HT2A (Fig. 3, B, left panel). This is in consistence with previous studies indicating a role for 5-HT2A in human Eos migration [11]. Pre-incubation of AML14.3D10 cells with inhibitors specific for ROCK (Y27632), MAPK (PD98059), PI3K (LY294002), PKC (BIM[I]) or calmodulin (TFP), a Ca^2+^-binding protein, prior to exposure to DOI resulted in significant inhibition of DOI-induced rolling on VCAM-1 relative to cells pretreated with DMSO (control) (Fig. 3, B, left and right panels). Pretreatment with PT, an inhibitor of G_αi_, had no effect on DOI-induced rolling (Fig. 3, B, right panel). The effect of DOI on the actin cytoskeleton was examined. Flow cytometry studies demonstrated that DOI induces actin polymerization in AML14.3D10 cells identified by increased FITC-phalloidin binding. DOI-induced actin polymerization was significantly inhibited by pre-treatment with MDL-100907 as well as Y27632, PD98059 and LY294002 (Fig. 3, C). Further, confocal microscopy studies with adherent AML14.3D10 cells (on rh VCAM-1) demonstrated that DOI-induced changes in cell morphology/cytoskeleton were markedly inhibited by MDL-100907, Y27632, PD98059 and LY294002 (Fig. 3, D). Cells treated with MDL-100907, PD98059 and LY294002 exhibited irregular spreading with few poorly formed filopodia, while treatment with the ROCK inhibitor Y27632 inhibited both cell spreading and development of filopodia. These studies indicate that rolling of AML14.3D10 cells induced by 5-HT or DOI involves 5-HT2A and activation of ROCK, MAPK, PI3K, PKC and calmodulin. Further, 5-HT2A activation with DOI induces actin polymerization and striking changes in the cytoskeletal/morphology of AML14.3D10 cells that are dependent on ROCK, MAPK and PI3K signaling and inhibited by the 5-HT2A-selective antagonist MDL-100907.

### DOI-induced trafficking of murine Eos involves ROCK, MAPK, PI3K, PKC and calmodulin

**Figure 4:**
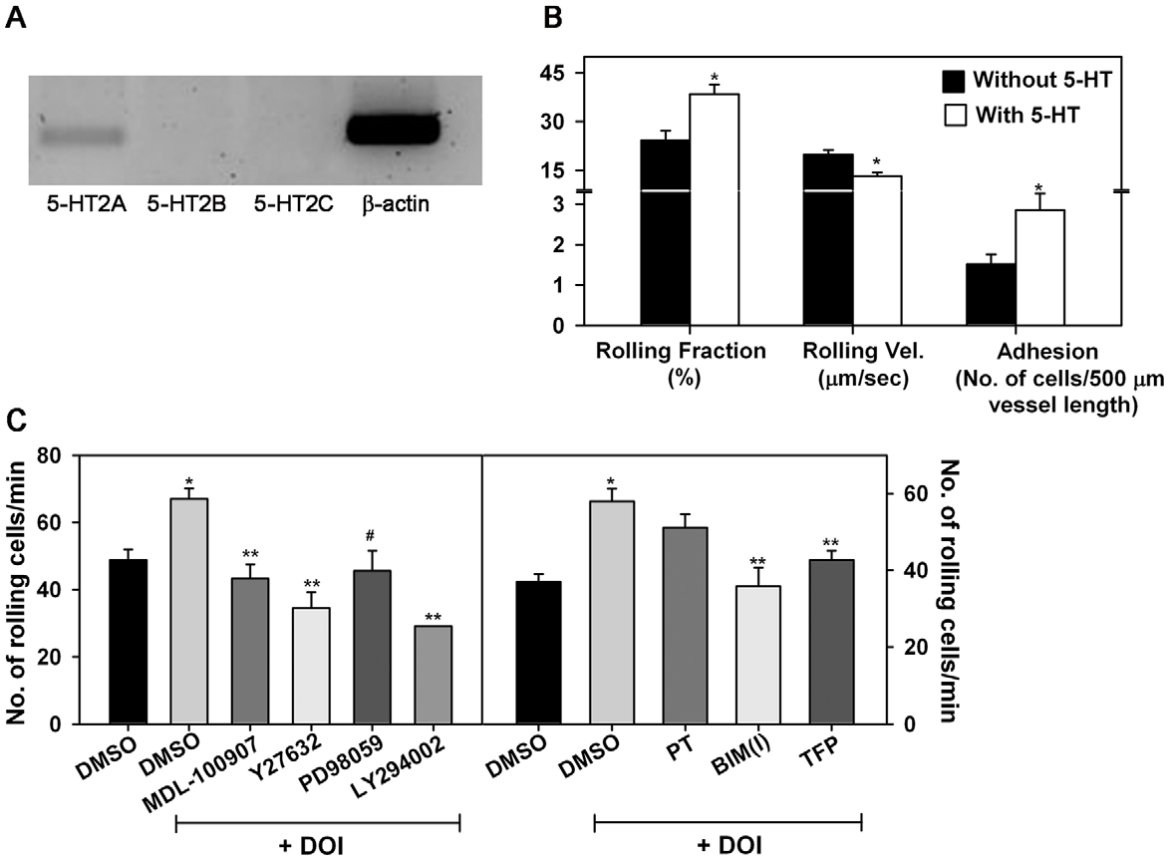
Effect of 5-HT and DOI on murine Eos rolling *in vivo* and *in vitro*. (**A**) Expression of 5-HT2 receptor subtypes by murine BM-Eos by RT-PCR. Expression of β-actin is shown as a control. (**B**) Trafficking of infused murine BM-Eos in inflamed (TNFα-stimulated) cremaster muscle microvessels of anesthetized mice before and after superfusion with 5-HT (100 nM) by IVM. The number of rolling cells is expressed as rolling fraction which is a percentage of the total number of cells passing through the same reference point. Rolling velocity of interacting Eos determined by off-line analysis of recorded video images by choosing four to six rolling Eos per venule and measuring the time taken for the cells to travel between two reference points (50-200 μm). Results represent mean rolling velocity of 143 cells before 5-HT treatment and 164 cells after 5-HT treatment. Number of Eos adhered in cremaster muscle microvessels of mice before and after treatment with 5-HT is also shown. An average of 16±2 recorded video images with 1–2 vessels per field were analyzed per mouse. Combined data (Mean ± SEM) of n = 3 mice/group is shown. *p<0.01 for comparison of Eos trafficking (rolling, rolling velocity and adhesion) before versus after 5HT. (**C**) Rolling of murine Eos on rm VCAM-1-coated cover-slips under conditions of flow *in vitro* after treatment with MDL-100907, Y27632, PD98059, LY294002 (left panel), BIM(I), TFP, PT (right panel) or DMSO (vehicle) as described in Figure 3. Combined data (Mean ± SEM) of 3 (right panel) –4 (left panel) independent experiments is shown. *p<0.01 for comparison of cells exposed to DOI versus vehicle. **p<0.01 and ^#^p<0.02 for comparison of cells exposed to DOI in the presence of inhibitor versus cells exposed to DOI in the presence of vehicle.

**Figure 5:**
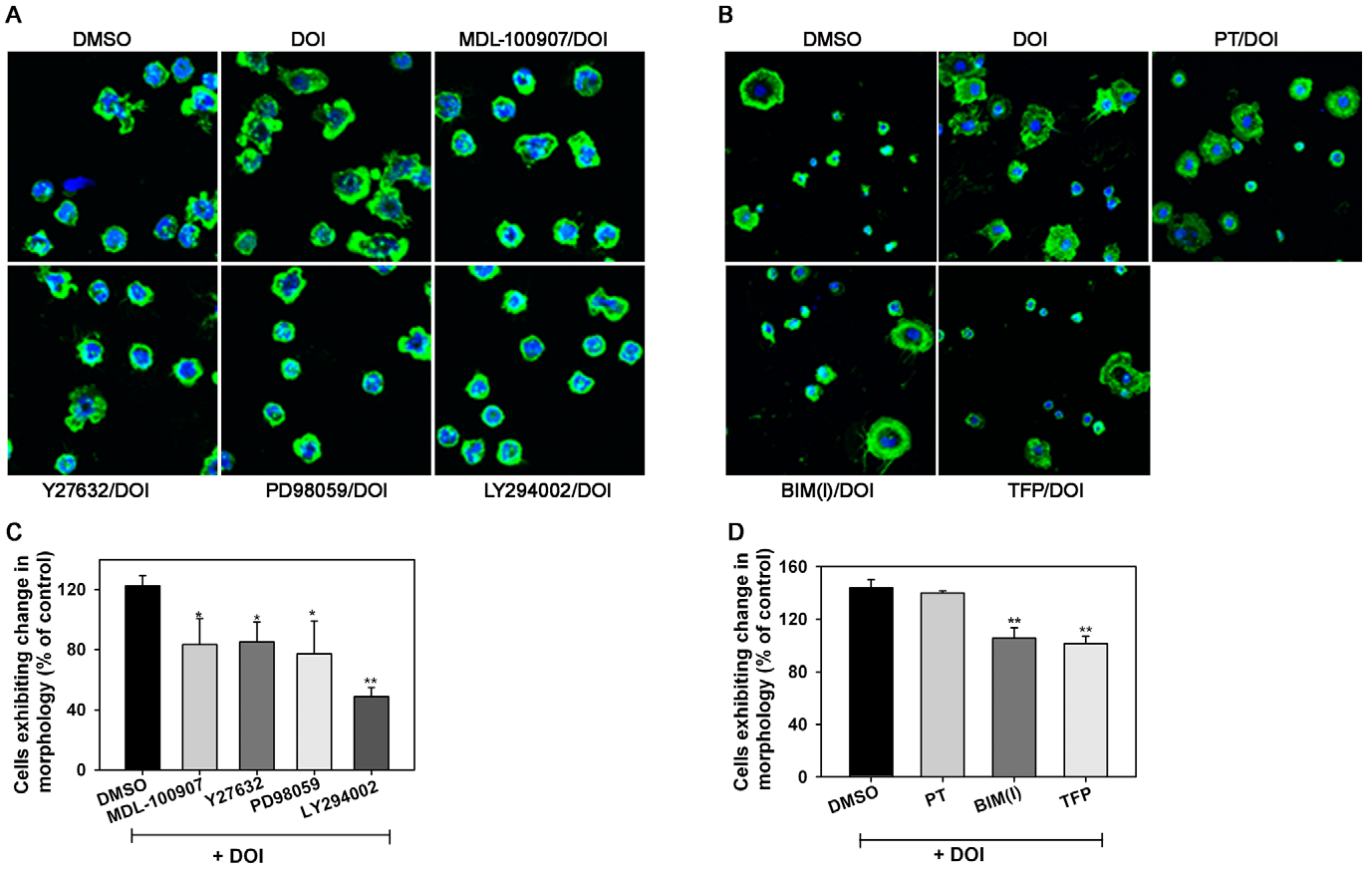
DOI induces changes in morphology of murine Eos. Effect of (**A**) MDL-100907, Y27632, PD98059, LY294002 as well as (**B**) PT, BIM(I) and TFP on DOI-induced changes in morphology of murine Eos adherent on rm VCAM-1 by confocal microscopy. Mouse Eos were allowed to adhere to rm VCAM-1-coated cover-slips in the presence of inhibitors or DMSO (vehicle) for 20 min and then exposed to 10 μM DOI for an additional 5 min prior to staining with FITC-phalloidin. Cells were counter-stained with DAPI. Magnification ×400. Quantitation of changes in morphology of murine Eos adherent on rm VCAM-1 exposed to DOI in the absence or presence of (**C**) MDL-100907, Y27632, PD98059, LY294002 as well as (**D**) PT, BIM(I) and TFP. Adhered cells in a fixed number of randomly selected non-overlapping fields of each cover-slip were counted and cells exhibiting cell spreading and/or distinct leading edges were identified and expressed as a percentage of the total number of cells in the field. Data shown in A and B is representative of three independent experiments with Eos from three different mice. Combined data (Mean ± SEM) from three independent experiments in C and two independent experiments in D in duplicate is shown. *p<0.05 and **p<0.01 for comparison of cells exposed to DOI in the presence of inhibitor versus cells exposed to DOI in the presence of vehicle.

Previous studies have demonstrated that airway recruitment of Eos in a mouse model of allergic inflammation can be inhibited by administration of cyproheptadine, a 5-HT2A inhibitor [11]. To understand how 5-HT may promote Eos recruitment *in vivo*, we first examined the effect of 5-HT on murine BM-Eos trafficking. RT-PCR with specific primers demonstrated that murine Eos express 5-HT2A but not 2B and 2C receptors (Fig. 4, A). *In vivo* studies in the cremaster muscle microcirculation by IVM revealed that infused murine Eos exhibit significantly increased rolling associated with a decrease in the velocities of the rolling Eos in microvessels under conditions of physiologic blood flow after superfusion of the microvessels with 5-HT compared to vehicle alone. Further, a significantly increased number of labeled BM-Eos were found adhered to the vessel walls after 5-HT-superfusion (Fig. 4, B). *In vitro*, activation of the 5-HT2A receptor with DOI significantly induced murine BM-Eos rolling on rm VCAM-1 relative to background rolling exhibited by cells exposed to DMSO alone (Fig. 4, C). DOI-induced rolling of murine BM-Eos was dependent on ROCK, MAPK, PI3K (Fig. 4, C, left panel), PKC and calmodulin (Fig. 4, C, right panel) since inhibitors/antagonists of these molecules inhibited rolling to near or below background levels observed with vehicle-treated cells. Blockade with PT which targets G_αi_ proteins did not alter DOI-induced Eos rolling (Fig. 4, C, right panel). In addition to inducing rolling, DOI induced distinct changes in morphology of BM-Eos adherent on VCAM-1 (Fig. 5, A). Several DOI-treated Eos exhibited spreading with formation of leading edges (Fig. 5, A, top row, center panel) while vehicle-treated control cells largely remained spherical with few cells spreading (Fig. 5, A, top row, left panel). Further, DOI-induced changes in murine BM-Eos morphology were prevented when cells were pretreated with MDL-100907 to inhibit 5-HT2A or with inhibitors of ROCK, MAPK, PI3K (Fig. 5, A), PKC and calmodulin (Fig. 5, B) but not of G_αi_ (Fig. 5, B). Quantitation of DOI-induced changes in morphology of adherent BM-Eos and their significant inhibition by pretreatment with MDL-100970 as well as inhibitors of ROCK, MAPK, PI3K, PKC and calmodulin is shown in Fig. 5, C and D. These data demonstrate that the effect of DOI on rolling under conditions of flow correlates with its effect on cell morphology. Further, murine Eos respond in a manner similar to AML14.3D10 cells when exposed to DOI. More importantly, together with the ability of 5-HT and DOI to alter the shape of non-adherent cells and induce actin polymerization, respectively, the above findings suggest that activation of 5-HT2A induces various cellular events in Eos that support directed movement.

### DOI-induced Eos migration is dependent on ROCK, MAPK, PI3K, PKC and calmodulin

**Figure 6:**
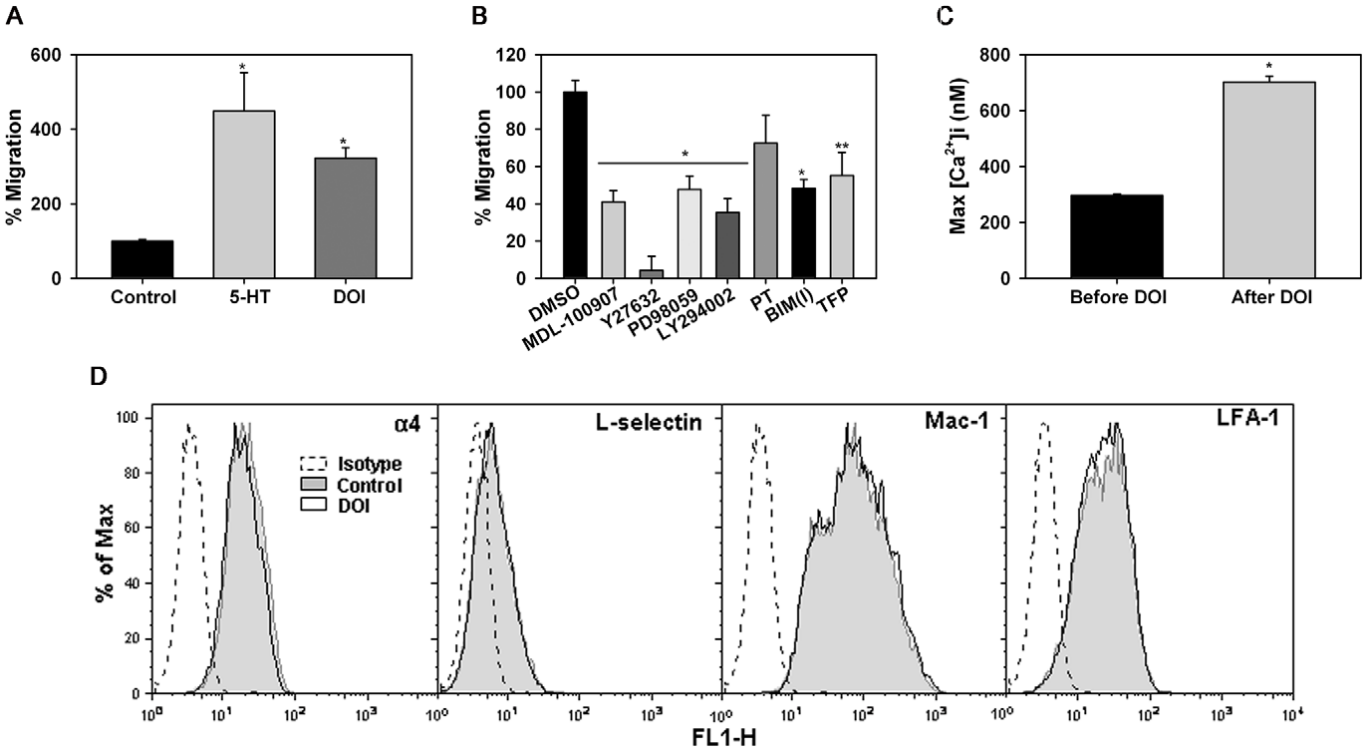
5HT and DOI induce migration of murine Eos. (**A**) Migration of murine Eos towards 10 μM 5-HT or DOI in Transwell® plates after 4 h at 37°C. The average number of cells/field/well was determined and results expressed as a percentage of background migration observed in wells containing medium alone. Combined data (Mean ± SEM) of 5 independent experiments is shown. *p<0.01 for comparison of 5-HT- or DOI-treated cells versus vehicle-treated cells. (**B**) Effect of MDL-100907, Y27632, PD98059, LY294002, BIM(I), TFP (all at 10 μM), and PT (100 ng/ml) on DOI-induced migration of murine Eos. Cells were pre-treated with inhibitors or DMSO (vehicle) alone for 20 min before addition to Transwell® Chambers. Results are expressed as a percentage of the migration of vehicle treated cells towards DOI. Combined data (Mean ± SEM) of 3 (for PT, BIM(I), TFP) or 5 (all other inhibitors) independent experiments in duplicate or triplicate is shown. *p<0.01 and **p<0.02 for comparison of cells treated with inhibitors versus cells treated with vehicle. (**C**) Basal and DOI-induced [Ca^2+^]*_i_* levels in murine Eos from 364 cells by digital videofluorescence imaging with Fura-2 AM. Representative data of three independent experiments performed in triplicate. *p<0.01 compared to unstimulated cells. (**D**) Expression of adhesion molecules by murine Eos after treatment with 10 μM DOI (or PBS) for 5 min by flow cytometry using rat mAbs against α4 (CD49), Mac-1 (CD11b) and LFA-1 (CD11a) followed by FITC-conjugated goat anti-rat IgG as the secondary antibody. Depending on the mAb, rat IgG2a or 2b was used as the isotype matched control. Expression of CD62L was evaluated using FITC-conjugated anti-mouse CD62L (BD Biosciences). FITC-conjugated rat-IgG2a was used as the isotype control for CD62L. All antibodies were used at a final concentration of 5 μg/ml. Data shown is representative of three independent experiments with Eos from different mice.

Since 5-HT is a known chemoattractant for human Eos and allergen-challenged mice treated with cyproheptadine (a 5-HT2A inhibitor) exhibit decreased Eos recruitment [11], we examined whether 5-HT and DOI induce murine Eos migration and how this is regulated. Similar to human Eos, 5-HT and DOI induced migration of murine BM-Eos *in vitro* (Fig. 6, A). Since ROCK, MAPK, PI3K, PKC and calmodulin were found to play a role in mediating DOI-induced Eos trafficking, we investigated their role in DOI-induced migration. Migration of murine BM-Eos in response to DOI was mediated via 5-HT2A since pretreatment of cells with MDL-100907 significantly inhibited migration. Further, blockade of ROCK, MAPK, PI3K, PKC and calmodulin significantly inhibited DOI-induced migration of murine Eos while inhibition of G_αi_ with PT marginally reduced DOI-induced migration of these cells that was not statistically significant (Fig. 6, B). In addition, exposure to DOI resulted in a significant increase in [Ca^2+^]_i_ (Fig. 6, C). Interestingly, migration of BM-Eos towards DOI was completely inhibited in the absence of Ca^2+^. While Eos showed a 1.8±0.15 fold increase in migration towards DOI relative to background in HBSS with Ca^2+^, they failed to migrate after intracellular Ca^2+^-depletion with BAPTA/AM in HBSS without Ca^2+^ suggesting an important role for Ca^2+^ in DOI-induced migration. Since 5-HT2A activation with DOI induced Eos trafficking and migration, the effect DOI on expression of α4, LFA-1, Mac-1, and L-selectin that are known to participate in Eos trafficking was investigated by flow cytometry. Treatment with DOI did not alter the level of expression of α4, LFA-1, L-selectin, or Mac-1 compared to vehicle treated cells (Fig. 6, D).

## Discussion

The importance of 5-HT in the pathogenesis of allergy and bronchial asthma was recognized as early as four decades ago [44], [45], [46], [47]. Since then, studies have clearly established a role for this molecule in promoting allergen-induced Eos recruitment, airway inflammation, AHR and remodeling [11], [24], [25], the hallmarks of allergic asthma, via interaction with the 5-HT2A receptor. However, the down-stream effects of 5-HT/5-HT2A interaction that regulate Eos trafficking and migration at a cellular level to promote recruitment during inflammation remain elusive. In this study, we evaluated the cellular mechanisms triggered when Eos are exposed to 5-HT in the context of recruitment.

*In vivo*, Eos recruitment to sites of inflammation is achieved by a multistep cascade involving the specific and sequential engagement of cell surface-expressed adhesion receptors with vascular counter ligands in inflamed blood vessels to initiate rolling on the vascular endothelium which is then followed by activation-dependent adhesion and chemokine-induced migration into inflamed tissues [48]. Using a 5-HT2A-selective antagonist (MDL-100907), our previous studies have demonstrated that 5-HT induces migration of human Eos in a 5-HT2A-dependent manner [11]. Current RT-PCR studies indicate that 5-HT2A is the predominant 5-HT receptor expressed by human Eos from allergic donors, although the level of expression of this receptor is variable among different donors. Circulating Eos are likely to be exposed to 5-HT since levels of this molecule are elevated in asthmatics [21]. Therefore, in addition to migration, 5-HT may even affect early events of cell trafficking such as rolling. Indeed *in vitro* flow chamber studies indicate that 5-HT as well as DOI, the 5-HT2A/2C selective agonist, induce rolling of human Eos on rh VCAM-1. In light of the donor-to-donor variation in the level of 5-HT2A expression, this effect of 5-HT and DOI on human Eos was confirmed using AML14.3D10 Eos-like cells which have consistent 5-HT2A expression. Further, murine BM-Eos which expressed only 5-HT2A but not 2B and 2C also demonstrated increased rolling on rm VCAM-1 *in vitro* when exposed to DOI in consistence with human Eos and AML14.3D10 cells. Since DOI is selective for 5-HT2A/2C receptors, and human as well as murine Eos did not appear to express 5-HT2C receptors, we anticipate that exposure of Eos to DOI promotes these effects via activation of 5-HT2A. *In vivo* studies with murine BM-Eos provide further support for the ability of 5-HT to promote not only increased rolling but also increased adhesion under conditions of physiologic blood flow within cremaster muscle microvessels.

Activation of cells triggers various cellular events including cytoskeletal reorganization, directed shape change and polarization that facilitate cell adhesion, spreading and migration [49], [50], [51], [52], [53]. Flow cytometry studies (GAFS assay) demonstrated that exposure of human Eos to 5-HT in suspension induces shape changes relative to untreated cells. Further, human Eos and AML14.3D10 cells adherent on VCAM-1 exhibited cell spreading with leading edges and distinct filopodia after 5-HT treatment. Thus, 5-HT can activate Eos to undergo changes in cell shape and morphology whether they are in circulation or interacting (adherent) with vascular adhesion molecules. It is conceivable that elevated 5-HT levels in serum during allergic asthma can initiate Eos rolling by inducing these changes that are essential for stable cell-cell interactions and directed movement. This notion is further supported by the ability of 5-HT to induce murine BM-Eos rolling and adhesion *in vivo*.

Most 5-HT receptors are known to couple to G-proteins [54]. When activated, G-proteins trigger various down-stream signaling cascades that initiate cell shape changes, cell spreading and formation of leading edge which are important for cell migration [55]. 5-HT2 receptors are coupled to G_αq/11_ proteins [16] and several studies have demonstrated that G_αq_-coupled receptors enhance motility by activating the classical PLC/PKC/calmodulin signaling axis (reviewed in [55]). Consistent with these findings, the PKC/calmodulin signaling pathway was found to play a role in Eos rolling (AML14.3D10 cells and murine BM-Eos) and migration (murine BM-Eos) induced by DOI. Further, DOI-induced rolling and migration of Eos was also found to be dependent on ROCK, MAPK and PI3K signaling but did not appear to involve G_αi_ proteins (not inhibited by PT). ROCK, MAPK and PI3K signaling was involved in DOI-induced actin polymerization in AML14.3D10 cells, and in addition to PKC and calmodulin, mediated DOI-induced changes in morphology of adherent murine BM-Eos. These studies suggest that stimulation of 5-HT2A activates multiple signaling pathways that trigger various cellular events which promote Eos trafficking and migration.

The Rho family of small GTP-binding proteins that activates ROCK [56] is known to modulate the actin cytoskeleton and participate in stress fiber formation [57]. Further, activation of RhoA and ROCK are essential for detachment of migrating leukocytes [58]. While up-stream activation of RhoA is largely mediated by receptor coupling to G_12/13_ proteins, studies have shown that G_αq/11_ can couple GPCRs to the rapid activation of RhoA, the activator of ROCK, independently of the PLC pathway [59]. Additionally, in NIH3T3 fibroblasts transformed to stably express 5-HT2A, stimulation of 5-HT2A receptors has been shown to activate the PLA2 signaling pathways via G_12/13_ coupling [60]. It is possible that when activated, 5-HT2A receptors expressed by Eos can couple to both G_αq/11_ and G_12/13_ and thus be able to mediate changes in the actin cytoskeleton and cell morphology via activation of ROCK. This is indeed the case with thrombin and bradykinin which promote the activation of Rho in a G_12/13_- as well as G_αq/11_-dependent fashion [55]. Studies have demonstrated that ROCK is an up-stream signal for MAPK activation during 5-HT-induced, 5-HT2-mediated migration of SMC [61]. In our studies, the 5-HT2A/2C agonist DOI induced phosphorylation of p-44/42 MAPK in AML14.3D10 cells and a role for MAPK in DOI-induced actin polymerization, changes in cell morphology and trafficking (rolling and migration) was clearly identified. However, it is not known whether MAPK activation is dependent or independent of ROCK activation. Further, there is also evidence for 5-HT2A-induced ERK (MAPK) activation independent of the PKC pathway via activation of Src and calmodulin [62] or by transactivation of tyrosine-kinase receptors such as fibroblast growth factor receptor 2 [63].

In addition to ROCK and MAPK, a role for PI3K was identified in mediating DOI-induced Eos trafficking and migration in the present study. Several studies indicate that PI3K functions as a down-stream signaling molecule for G_αi_-coupled receptors such as 5-HT1 [54]. The lack of inhibition of Eos rolling and migration in the presence of PT in our studies suggest that G_αi_ proteins are not likely to be involved in these DOI-induced 5-HT2A-mediated events. More recent studies have demonstrated a role for PI3K in 5-HT2-mediated events as well. 5-HT-induced proliferation of pulmonary artery SMCs was found to be mediated by 5-HT2 in a PI3K-dependent manner [64]. Since 5-HT receptors can transactivate tyrosine kinase receptors [54] which signal via PI3K, this may be an alternate pathway by which PI3K mediates the effects of 5-HT2A. There is also the possibility of a still unidentified pathway connecting G_αq/11_-activated PLC pathway to PI3K upon 5-HT2A stimulation. Exposure of murine Eos to DOI was found to increase levels of [Ca^2+^]_i_ and cells failed to migrate towards DOI in the absence of Ca^2+^ (intracellular and extracellular). Ca^2+^ is a biologically important second messenger which plays an important role in regulation of events critical for cell migration such as cytoskeleton dynamics, cell adhesion and cell migration [50]. 5-HT has been shown to induce an increase in [Ca^2+^]_i_ in both airway epithelial cells [20] as well as AM [5] via activation of 5-HT2 receptors. Activation of G_αq_-coupled receptors (like 5-HT2A) is associated with activation of PLC followed by a series of signaling events resulting in increased [Ca^2+^]_i_ [65], which in turn is important for regulation by down-stream Ca^2+^-dependent effectors such as calmodulin [66]. Indeed, calmodulin was found to be essential for not only promoting DOI-induced changes in morphology of adherent Eos but also their ability to roll and migrate in the present study.

Eos rolling is largely mediated by α4β1-VCAM-1 interactions [67]. While DOI induced murine BM-Eos rolling, it did not appear to have any effect on the level of expression of α4 integrins, LFA-1, L-selectin or Mac-1. Recent studies with stromal derived factor-1α-treated monocytes have identified a critical role for PLC/Ca^2+^/calmodulin signaling pathway in altering the conformation of α4 integrins to a high-affinity conformation [68]. Since DOI-induced Eos rolling and migration requires PLC and calmodulin, activation of this signaling pathway by DOI-5-HT2A interaction may support Eos trafficking by altering the conformation of α4. However, additional studies are needed to confirm this. In summary, stimulation of 5-HT2A by 5-HT (or DOI) induces Eos trafficking and migration by increasing [Ca^2+^]_i_ levels and promoting actin polymerization associated with changes in cell shape/morphology. These 5-HT2A-mediated pro-migratory events appear to involve ROCK, MAPK and PI3K in addition to PKC and calmodulin. These studies also demonstrate common signaling pathways regulating 5-HT-mediated trafficking and migration in both human and murine Eos.
